# 阿米福汀在局部晚期非小细胞肺癌放疗中作用的*meta*分析

**DOI:** 10.3779/j.issn.1009-3419.2012.09.06

**Published:** 2012-09-20

**Authors:** 升晔 王, 沂平 张, 苏展 张, 胜林 马

**Affiliations:** 1 310009 杭州，浙江大学医学院附属第二医院肿瘤科 Department of Oncology, the Second Affiliated Hospital of Zhejiang University, Hangzhou 310009, China; 2 310022 杭州，浙江省肿瘤医院化疗科 Department of Chemotherapy, Zhejiang Cancer Hospital, Hangzhou 310022, China; 3 310006 杭州，杭州市第一医院肿瘤科 Department of Oncology, the First Hospital of Hangzhou, Hangzhou 310006, China

**Keywords:** 阿米福汀, 肺肿瘤, 放疗, *meta*分析, Amifostine, Lung neoplasms, Radiotherapy, *Meta*-analysis

## Abstract

**背景与目的:**

阿米福汀（Amifostine）是否影响非小细胞肺癌（non-small cell lung cancer NSCLC）放疗疗效并降低放疗相关副反应一直存在较大争议，本研究旨在探讨阿米福汀在局部晚期NSCLC放疗近期疗效及预防放疗副反应中的作用。

**方法:**

检索Medline、CENTRAL（the Cochrane central register of controlled trials）、EMBASE、中国生物医学文献数据库系统（CBM）、中国期刊全文数据库（CNKI）、万方数据库、美国临床肿瘤学会（ASCO）、欧洲肿瘤协会（EMSO）官方网等，检索公开发表的有关局部晚期NSCLC放疗期间应用阿米福汀的随机临床对照研究。应用Stata 11.0统计软件分析阿米福汀对放疗近期疗效及副反应发生率的影响。

**结果:**

最终纳入9项研究，阿米福汀组患者381例，对照组388例。涉及近期疗效的研究8项，阿米福汀组患者328例，对照组333例。结果显示放疗期间接受阿米福汀治疗的患者完全缓解（complete response, CR）、部分缓解（partial response, PR）和客观缓解（objective response, OR）的相对危险度（relative risk, RR）分别为1.16（95%CI: 0.90-1.50, *Z*=1.07, *P*=0.29）、1.02（95%CI: 0.87-1.19, *Z*=0.21, *P*=0.83）和1.06（95%CI: 0.97-1.17, *Z*=1.31, *P*=0.20）。涉及放疗副反应研究7项，阿米福汀组患者367例，对照组371例，放疗期间接受阿米福汀治疗的患者3级-4级放射性食管炎和放射性肺炎发生的RR分别为0.51（95%CI: 0.37-0.72, *Z*=3.88, *P* < 0.001）和0.51（95%CI: 0.26-0.99, *Z*=1.98, *P*=0.04）。

**结论:**

阿米福汀可以降低NSCLC放疗中放射性食管炎和放射性肺炎的发生率，但不降低放疗的近期疗效。

## Abstract (en)

**Background and objective:**

Controversy exists on whether amifostine can reduce the efficacy and decrease the side effects of non-small cell lung cancer (NSCLC) treated by radiotherapy. The aim of this *meta*-analysis is to evaluate the efficacy and side effects of amifostine in NSCLC patients treated with radiotherapy.

**Methods:**

Open published randomized controlled trials on the efficacy and side effects of amifostine in NSCLC patients treated with radiotherapy were collected from Medline, Cochrane Central Register of Controlled Trials, EMBSE, CBM, CNKI, WANFANG, American Society of Clinical Oncology, and European Society of Medical Oncology databases. The pooled efficacy and side effects of amifostine in these patients were calculated using the statistics software Stata 11.0.

**Results:**

Nine trials that included 769 (381 and 388 in each arm) patients were analyzed. The pooled relative risk of complete, partial, and objective responses were 1.16 (95%CI: 0.90-1.50, *Z*=1.07, *P*=0.29), 1.02 (95%CI: 0.87-1.19, *Z*=0.21, *P*=0.83) and 1.06 (95%CI: 0.97-1.17, *Z*=1.31, *P*=0.20), respectively. The side effects in seven trials including 738 (367 and 371 in each arm) patients were analyzed. The pooled relative risk of developing grades 3 to 4 esophagitis and pneumonitis were 0.51 (95%CI: 0.37-0.72, *Z*=3.88, *P* < 0.001) and 0.51 (95%CI: 0.26-0.99, *Z*=1.98, *P*=0.04), respectively.

**Conclusion:**

Amifostine can significantly decrease the risk of developing serious esophagitis and pneumonitis without reducing the response rate in NSCLC patients treated by radiotherapy.

目前肺癌已经成为全球范围内发病率和死亡率最高的恶性肿瘤，全球每年因肺癌而死亡的病例高达120万^[1]^，肺癌已经成为威胁公共健康的重大问题。肺癌患者确诊时80%已为晚期或局部晚期，大多失去了手术机会，往往需要采取化疗和放疗。阿米福汀目前已经被美国食品药品监督管理局（FDA）批准用于减轻头颈部放疗引起的毒副反应，但其应用是否可以降低非小细胞肺癌（non-small cell lung cancer, NSCLC）患者放疗期间严重的放射性食管炎和放射性肺炎的发生率，及是否会降低放疗疗效一直存在争议^[2, 3]^。本研究采用*meta*分析的方法对阿米福汀在NSCLC放疗中的作用进行探讨。

## 1 材料与方法

### 1.1 文献检索

检索公开发表的关于NSCLC放疗期间应用阿米福汀的随机临床对照研究。检索语种为英语和汉语。以“Amifostine[MeSH]/Amifostine[text]; Non-small cell lung cancer (NSCLC)[MeSH]/Non-small cell lung cancer(NSCLC)[text]; Non small cell lung neoplasm[MeSH]/ Non small cell lung neoplasm[test] AND RCT/Randomized controled trials; random^*^”检索Medline、CENTRAL、EMBASE、ASCO、EMSO等英文数据库；以“阿米福汀/氨磷汀；非小细胞肺癌；NSCLC”检索CNKI、CBM、万方等中文数据库，同时进一步对入选文献的参考文献进行扩大检索以发现可能合格的文献。

### 1.2 研究的筛选

#### 1.2.1 入选标准

① 研究设计：随机临床对照试验，无论是否采用盲法；②研究对象：局部晚期非小细胞肺癌接受放疗患者；③干预措施：静脉或皮下应用阿米福汀；④对照组：放疗同时给予安慰剂或观察。

#### 1.2.2 排除标准

① 小细胞肺癌患者的研究；②非前瞻性随机对照等回顾性研究；③放疗期间应用其它药物的患者；④脏器功能不全患者；⑤原文未提足够的资料用以计算相对危险程度（relative risk, RR）。

#### 1.2.3 结局指标

疗效指标：根据WHO制定的实体肿瘤近期疗效评价指标，完全缓解（complete response, CR）：全部病灶消失维持4周；部分缓解（partial response, PR）：瘤体缩小30%-50%维持4周；客观缓解（objective response, OR）：完全缓解+部分缓解。毒副反应指标：根据放射治疗协作组（RTOG）制定的放疗相关毒性反应标准^[4]^评价3级-4级放射性食管炎、放射性肺炎发生率。

### 1.3 纳入研究质量评价

Cochrane手册RCT质量评价标准（http://www.cochrane.org/search/site/handbook）中的随机化、隐蔽分组、盲法、结果数据的完整性、选择性报告研究结果和与其它偏倚来源进行评价。对应每一条标准，文献中明确满足则为“+”；不清楚为“？”；不满足或未提及则为“-”。对每一篇文献的质量评价采取双人平行评价的方法。

### 1.4 数据提取

双人平行提取：①一般资料：文章题目、作者、发表日期、期刊名称；②研究特征：患者一般情况、临床基线可比性、干预方法；③文献质量:随分组、盲法（双盲/单盲）、隐蔽分组、意向性分析、统计方法描述；④结局指标：近期疗效完全缓解、部分缓解和总缓解率，放射性食管炎、放射性肺炎发生率等。

### 1.5 统计方法

该*meta*分析采用近期疗效完全缓解、部分缓解和总缓解率的相对危险度RR和放射性食管炎、放射性肺炎发生率的相对危险度RR为效应指标，并以95%CI表示，双侧*P* < 0.05认为差异有统计学意义。异质性检验采用*Q*统计量的*I*^2^检验分析，双侧*P* > 0.05不存在明显的统计学异质性，数据合并采用固定效应模型（fixed effect model）；*P*≤0.05认为存在较大异质性，此时进一步分析其异质性的来源，并进行亚组分析或采用描述性分析。异质性因素单纯由统计学引起，而无临床或方法学异质性则采用随机效应模型（random effect model）进行数据分析。统计分析采用Stata 11.0软件完成。

## 2 结果

### 2.1 检索结果

初检文献获得211篇，阅读标题和摘要剔除明显不符合要求文献187篇，阅读全文剔除文献15篇，最终有9项研究^[2, 3, 5-11]^纳入评价（图 1），其中8项研究纳入放疗近期疗效，阿米福汀组患者328例，对照组333例；7项研究纳入放疗副反应，阿米福汀组患者367例，对照组371例。9篇文献中7篇为英文文献，2篇为中文文献，原始研究提供了较为完整的数据资料（表 1）。依据Cochrane手册RCT质量评价标准对纳入的研究进行方法学质量评价，评价结果具体见图 2。

**1:**
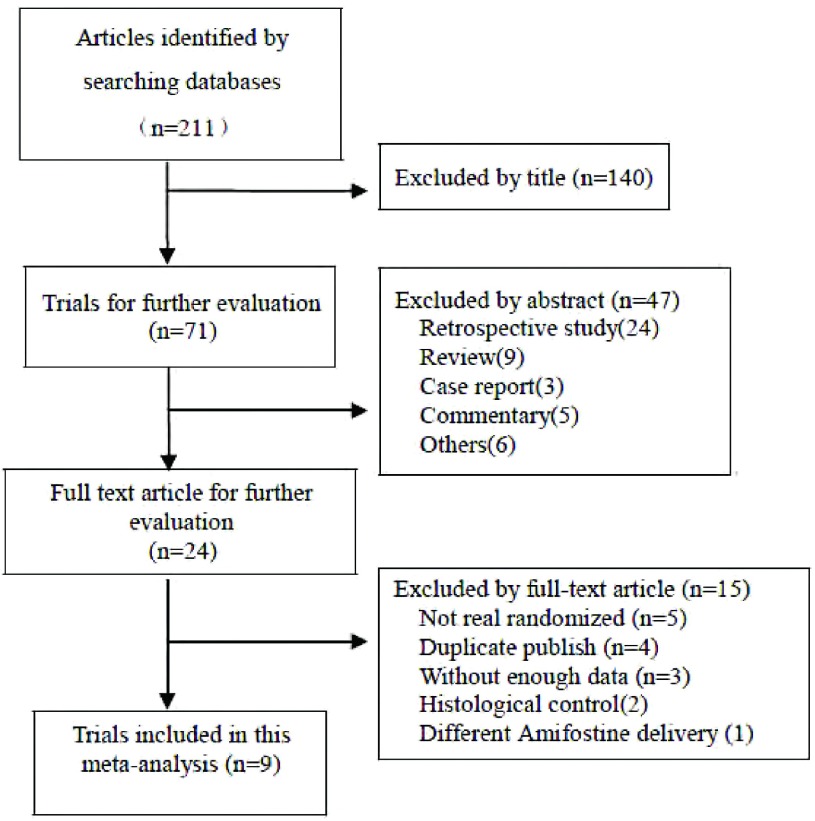
纳入研究的流程图 The flow chart of literature search and study selection

**1:** 纳入研究的基本特征 The general characteristics of included studies

Author	Amifostine/Control (*n*)	Amifostine dose	Administration	Radiotherapy	Year
Koukourakis^[2]^	19/17	500 mg/d	IH	64 Gy, 2 Gy qd	2000
Antonadou^[3]^	44/53	340 mg/m^2^•d	Ⅳ	55 Gy-60 Gy, 2 Gy qd	2001
Senzer^[5]^	24/25	200/m^2^•d	Ⅳ	64.8 Gy, 1.8 Gy qd	2002
Antonadou^[6]^	36/32	300/m^2^•d	Ⅳ	55 Gy-60 Gy, 2 Gy qd	2003
Leong^[7]^	30/30	740 mg/m^2^•d	Ⅳ	60 Gy-66 Gy, 2 Gy qd	2003
Komaki^[8]^	31/31	500 mg, 2 times/wk	Ⅳ	69.6 Gy, 1.2 Gy bid	2004
Movsas^[9]^	114/115	500 mg 4 times/wk	Ⅳ	69.6 Gy, 1.2 Gy bid	2005
Weng ^[10]^	30/30	300/m^2^•d	Ⅳ	50 Gy-60 Gy, 2 Gy qd	2007
Li^[11]^	53/55	200/m^2^•d	Ⅳ	60 Gy-66 Gy, 2 Gy qd	2010

**2:**
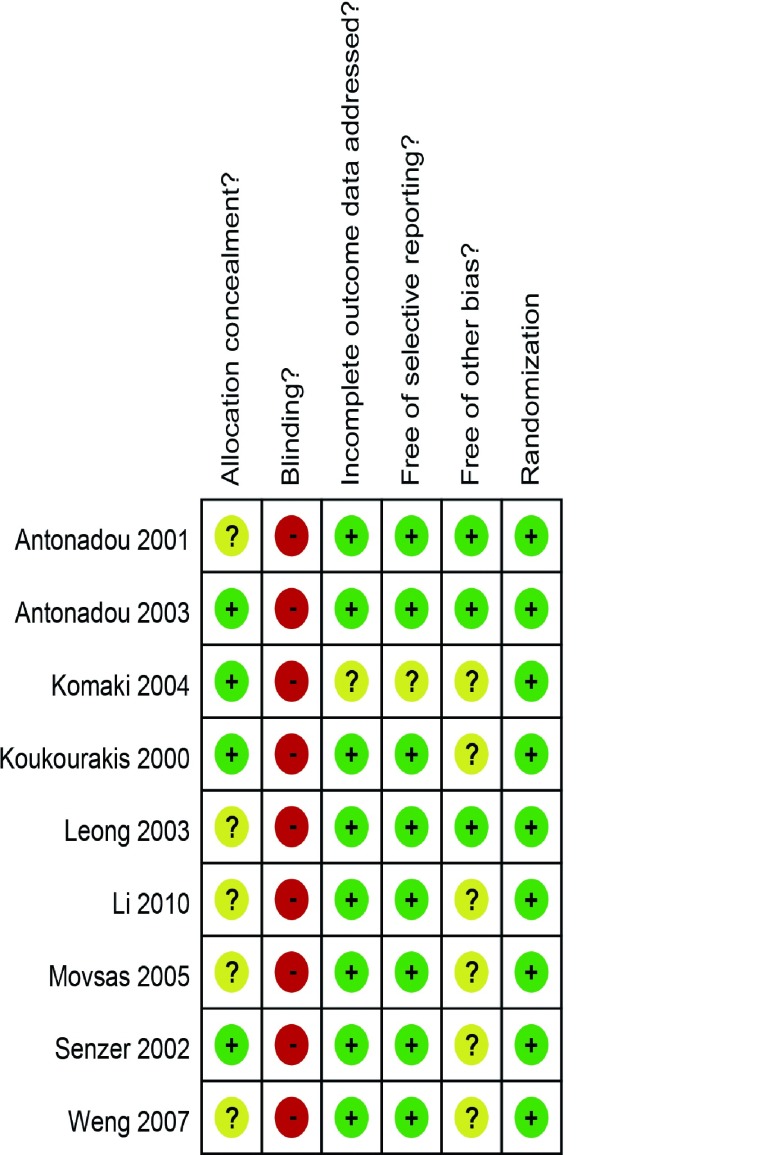
纳入研究的质量评价 The evaluation of included studies. (+ equals "low risk"; - equals "high risk"; ? equals "moderate risk").

### 2.2 异质性检验

分别以CR、PR、OR、放射性食管炎和放射性肺炎发生率相对危险度RR为效应指标，行统计学异质性检验。CR、PR、OR等指标无统计学异质性（*P* > 0.05），合并分析采用固定效应模型；放射性食管炎和放射性肺炎，存在统计学异质性（*P* < 0.05），合并分析采用随机效应模型。

### 2.3 数据合成

涉及近期疗效的研究8项，阿米福汀组患者328例，对照组333例。结果显示放疗期间接受阿米福汀治疗的患者CR、PR、和OR的相对危险度RR分别为1.16（95%CI: 0.90-1.50, *Z*=1.07, *P*=0.29），1.02（95%CI: 0.87-1.19, *Z*=0.21, *P*=0.83），1.06（95%CI: 0.97-1.17, *Z*=1.31, *P*=0.20）（图 3）；涉及放疗副反应研究7项，阿米福汀组患者367例，对照组371例，放疗期间接受阿米福汀治疗的患者放射性食管炎和放射性肺炎发生的RR分别为为0.51（95%CI: 0.37-0.72, *Z*=3.88, *P* < 0.001）和0.51（95%CI: 0.26-0.99, *Z*=1.98, *P*=0.04）（图 4）。

**3:**
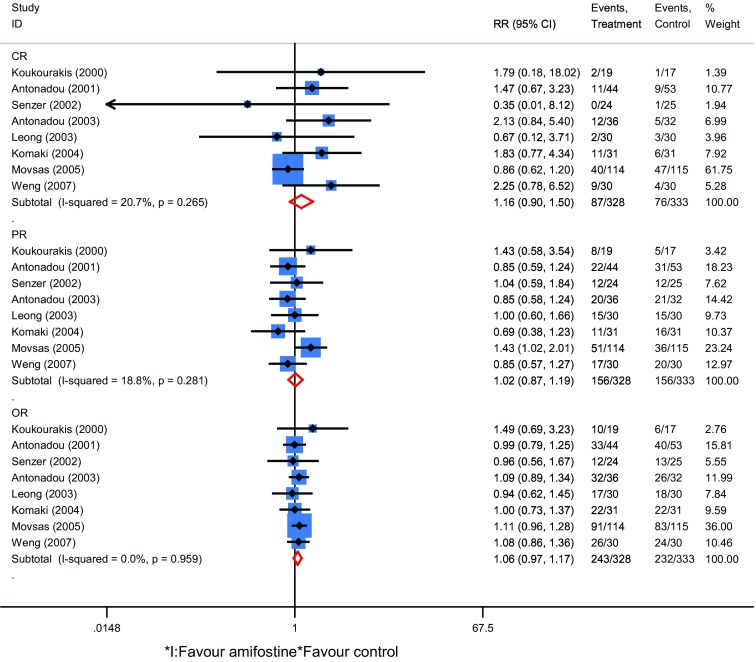
近期疗效的森林图 Forest plot of response rate for NSCLC. NSCLC: non-small cell lung cancer.

**4:**
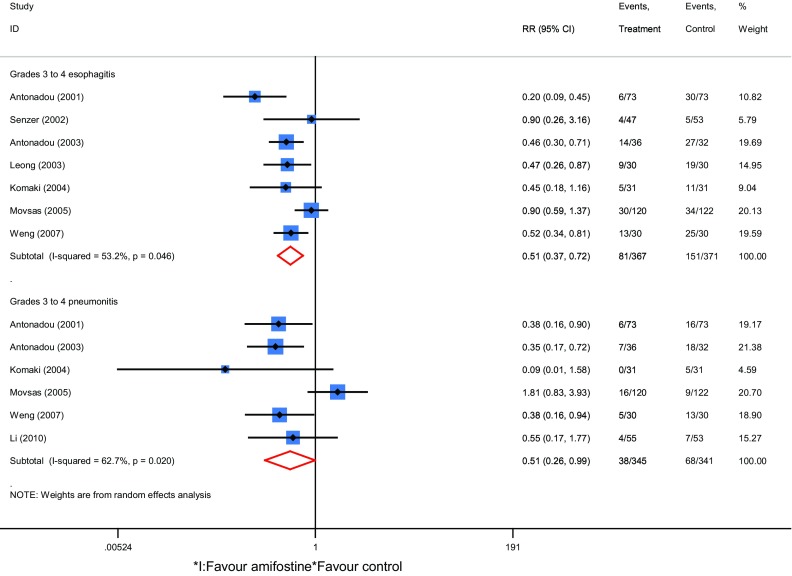
放疗副反应的森林图 Forest plot of side effect for NSCLC

### 2.4 发表偏倚的

Begg’test中Pr > |z|=0.18 > 0.05，图中代表各研究的点沿中间水平线向两侧均匀分布，且位于预计95%置信区间内（图 5），提示不存在明显的发表偏倚。

**5:**
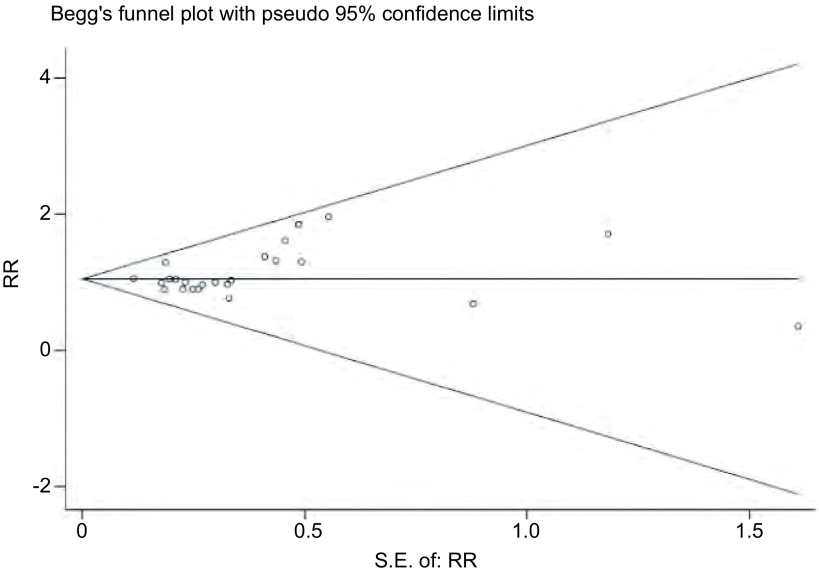
*Begg*法检测发表偏倚的漏斗图 The funnel plot of publication bias

## 3 讨论

目前，肺癌在全球范围内高发，每年因肺癌而死亡的病例高达100多万^[12]^。近年来我国肺癌的发病率呈逐渐上升趋势，预计到2015年我国将成为全球肺癌发病人数最多的国家^[13]^。肺癌患者确诊时80%已为晚期或局部晚期，大多失去了手术机会，往往需要行放化疗。肺癌的放疗疗效呈现剂量依赖性，高剂量的放疗可以提高肿瘤的缓解率但高剂量放疗同时会带来相关的严重副反应，如严重的放射性食管炎和放射性肺炎。

阿米福汀是上世纪50年代冷战时期美国Walter Reed陆军研究院研制的一种预防核辐射保护剂，当时用于保护美军在核战争中减少核辐射的伤害药理学研究^[14]^显示，阿米福汀可以被组织中与细胞膜结合的碱性磷酸酶水解脱磷酸成为具有活性的WR-1065，其巯基结构可以清除组织中的氧自由基，因而能减低顺铂等的毒性。随后的研究发现，该药物可以降低某些肿瘤放疗中的毒副反应，因此，该药物已被美国FDA批准用于非小细胞肺癌和卵巢癌使用顺铂引起的肾脏毒性，同时也被批准用于降低或预防头颈部放疗引起的口腔粘膜干燥。但关于该药物用于预防或减轻胸部肿瘤放疗中出现的不良反应和是否降低放疗疗效方面的研究结论并不一致。Komaki^[8]^及其同事研究发现，肺癌放疗过程中加用阿米福汀可以使严重的放射性食管炎和放射性肺炎的发生率降低19%和16%。Antonadou等^[3]^的研究也得到了相似的结果，在他们的研究中阿米福汀组严重放射性食管炎和放射性肺炎的发生率仅为对照组的1/5和1/3，显示出了阿米福汀良好的疗效。但随后Senzer等^[5]^和Movsas等^[9]^分别进行的两项随机临床对照研究发现阿米福汀对肺癌放疗过程中严重的放射性食管炎和放射性肺炎并无预防和保护作用。因此，阿米福汀是否可以预防和降低胸部放疗中放射性食管炎和放射性肺炎发生的问题存在较大争议。关于放疗过程中应用阿米福汀是否会降低放疗疗效的问题研究结果也存在一定的差异。大多数研究结果^[2, 3, 5]^认为，阿米福汀的应用并不会降低放疗的近期疗效，但Movsas等^[9]^的一项较大样本的Ⅲ期临床对照研究结果却提示阿米福汀的应用可能会降低肺癌放疗的缓解率。

本研究结果共纳入9项研究，阿米福汀组患者381例，对照组388例，涉及近期疗效的研究8项，阿米福汀组患者328例，对照组333例，结果显示阿米福汀并不降低肺癌放疗的近期疗效；涉及放疗副反应的研究7项，阿米福汀组患者367例，对照组371例，阿米福汀可以显著降低肺癌放疗中严重的3级-4级食管炎和放射性肺炎发生率。本*meta*分析中纳入的研究人群均为晚期或局部晚期的NSCLC患者，各研究中受试人群的临床异质性不明显，近期疗效评价中各研究间不存在明显的统计学异质性，且采用固定效应模型，因此对于阿米福汀并不降低肺癌放疗近期疗效的结论应较为肯定。但本研究也存在一定的局限性：①阿米福汀是否降低肺癌放疗中3级-4级放射性食管炎和放射性肺炎发生危险的分析结果存在明显的统计学异质性，采用随机效应模型进行计算，其结论稳定性可能受到一定的影响^[14]^；②纳入研究的文献中均未准确提及盲法，可能对结果造成一定的偏倚。

虽然本*meta*分析和大多数随机临床对照研究结果均支持阿米福汀在降低肺癌放疗中严重食管炎和肺炎发生率的同时并不降低肺癌放疗的近期疗效，似乎该药物只对正常组织起保护作用，而对肿瘤细胞无保护作用。分析原因可能与该药物在肿瘤细胞和正常细胞中的分布或药理作用并不完全相同有关^[15]^。正常组织细胞膜结合碱性磷酸酶的能力远远高于肿瘤组织。此外，阿米福汀在正常组织中的吸收为主动运输，而在肿瘤组织中为被动扩散，且正常组织的中性pH环境比某些肿瘤组织的酸性pH环境更有利于阿米福汀的吸收。因此，阿米福汀在正常组织内的浓度可能要远远高于肿瘤组织^[16]^。但关于该药物在肿瘤细胞和正常细胞中是否存在不同的药理作用尚无确切证据。

在过去的数十年中，临床上尤其是肿瘤放化疗中针对阿米福汀进行了广泛而深入的研究，多数研究结果显示阿米福汀是具有选择性的广谱细胞保护剂，在肿瘤的临床治疗中起着重要的作用^[16, 17]^。但关于阿米福汀在NSCLC治疗中更深层次的作用，如应用阿米福汀后患者的长期疗效总生存时间、疾病无进展生存时间等肺癌治疗的更重要的临床指标方面有待进一步研究。因此，需要设计优良的大样本临床随机对照试验对阿米福汀更多相关疗效进行进一步深入研究。
